# Intraring allostery controls the function and assembly of a hetero-oligomeric class II chaperonin

**DOI:** 10.1096/fj.201701061R

**Published:** 2018-01-05

**Authors:** Deborah K. Shoemark, Richard B. Sessions, Andrea Brancaccio, Maria Giulia Bigotti

**Affiliations:** * School of Biochemistry, University of Bristol, Bristol, United Kingdom;; † Istituto di Chimica del Riconoscimento Molecolare–Consiglio Nazionale delle Ricerche (CNR), Università Cattolica del Sacro Cuore, Rome, Italy

**Keywords:** molecular chaperones, protein folding, molecular dynamics

## Abstract

Class II chaperonins are essential multisubunit complexes that aid the folding of nonnative proteins in the cytosol of archaea and eukarya. They use energy derived from ATP to drive a series of structural rearrangements that enable polypeptides to fold within their central cavity. These events are regulated by an elaborate allosteric mechanism in need of elucidation. We employed mutagenesis and experimental analysis in concert with *in silico* molecular dynamics simulations and interface-binding energy calculations to investigate the class II chaperonin from *Thermoplasma acidophilum*. Here we describe the effects on the asymmetric allosteric mechanism and on hetero-oligomeric complex formation in a panel of mutants in the ATP-binding pocket of the α and β subunits. Our observations reveal a potential model for a nonconcerted folding mechanism optimized for protecting and refolding a range of nonnative substrates under different environmental conditions, starting to unravel the role of subunit heterogeneity in this folding machine and establishing important links with the behavior of the most complex eukaryotic chaperonins.—Shoemark, D. K., Sessions, R. B., Brancaccio, A., Bigotti, M. G. Intraring allostery controls the function and assembly of a hetero-oligomeric class II chaperonin.

Chaperonins are large (≈10^6^ Da) oligomeric complexes that aid in the folding of nonnative polypeptides that are either newly synthesized or misfolded as a consequence of physiologic or pathologic cellular processes. They fulfill this crucial role in all living organisms by sequestering, through a series of ATP-driven and highly controlled allosteric conformational rearrangements, nonnative polypeptides in a central cavity where they can fold unperturbed by the cellular environment (1, 2). Chaperonins are composed of two multisubunit rings stacked back-to-back to form a toroidal cylinder enclosing an internal space, often referred to as the folding chamber. This architecture is common to chaperonins from eubacteria and cell organelles (class I) and those from archaea and the eukaryotic cytosol (class II). The latter group is structurally more complex than its bacterial counterpart, mostly owing to heterogeneity in subunit composition. The type of subunits that form each 8- [occasionally 9- (3)] membered ring ranges from the 1–3 found in archaebacterial chaperonins, or thermosomes, to the 8 different subunits of eukaryotic chaperonin (CCT)/TriC (for chaperonin containing TCP-1/TCP-1 ring complex). Despite some degree of sequence variability, the high-resolution crystal and cryo-electron microscopy (cryo-EM) structures of the archaeal (4–6) and eukaryotic (7–9) class II chaperonins reveal that all subunits share an identical domain organization. This consists of an equatorial domain that forms the interring interface and contains the ATP-binding site (an apical domain responsible for the interaction with nonnative substrates and containing most of the intraring contacts between subunits) and an intermediate domain, which connects the other two and acts as a hinge that is fundamental for the propagation of allosteric signals. Furthermore, a helical module at the tip of the apical domain acts as a lid, closing the folding chamber during ATP hydrolysis.

Despite considerable advances made in recent years, many of the structural and functional details behind type II chaperonin activity and their relation to the allosteric behavior of these ATPase machines are still poorly understood. This is mainly due to the intrinsic structural complexity of class II chaperonins, whose hetero-oligomeric nature makes a recombinant approach particularly challenging. As a matter of fact, Yamamoto *et al.* (10) only recently succeeded in producing sufficiently large amounts of CCT from a thermophilic fungus in *Escherichia coli* for recombinant analysis. Indeed, most of the information available to date comes from *in vitro* studies conducted on CCT purified from mammalian and yeast cells. In a pioneering study, ATP-binding or -hydrolysis site–directed mutants of different subunits of CCT from *Saccharomyces cerevisiae* highlighted a hierarchy in ATP binding and hydrolysis among subunits possibly independent of their intrinsic structural role in the oligomer (11, 12). Such a complex scenario has been corroborated by a series of mutagenesis studies on *S. cerevisiae* CCT (13–15) showing a gradient of ATP affinities in the different subunits, so that subunits with similar affinities for ATP group together on the opposite sides of each ring. This has been confirmed by cryo-EM analysis of a newly identified state, partially preloaded with nucleotide, and of the ATP-bound state (16). Further analysis of the data on ATP-site mutants led to the proposal of a model describing an intraring sequential allosteric mechanism of ATP hydrolysis by CCT (17). It has been speculated, based on the differential characteristics and behavior of the subunits, that such a complex mechanism could increase the efficiency of folding of multidomain proteins by allowing their release from the chaperonin, domain by domain, in a sequential order (15, 17–19).

Thermosomes are well placed to provide further insight into this phenomenon, as their reduced subunit heterogeneity correlates well with the lower abundance of multidomain proteins in the archaeal proteome compared with their eukaryotic counterparts (20, 21).

Despite their lower complexity in subunit composition, hetero-oligomeric thermosomes present similarities with the allosteric behavior of CCT (22–24). Indeed, it has been shown that the thermosome from the acidophilic archaeon *Thermoplasma acidophilum* (*Ta*) thermosome, a hexadecamer composed of α and β subunits, has a biphasic ATPase activity, indicative of the presence of 2 different classes of ATP-binding sites (22, 25).

The *Ta* thermosome has been taken as a model for class II chaperonin studies (22, 25–28) because it was the first of such proteins whose structure was solved at high resolution by X-ray crystallography (4). It is composed of two octameric rings of alternating α and β subunits. Our recombinant system for the *Ta* thermosome employs an intraloop His_6_-tag in each α-subunit and allows for a carefully balanced overexpression of α and β subunits in *E. coli*. This system produces unprecedented amounts of functional αβ-thermosome hexadecamers (29) and has served well for the efficient expression and purification of mutants for this study. A panel of single residue mutants was chosen aimed at impairing either ATP binding or hydrolysis in the α and β subunits individually. Experiments were devised to explore whether each mutant altered ATP binding or hydrolysis in the system, influenced subunit heterogeneity in thermosome assembly affected the refolding behavior and allowed us to begin to pinpoint the specific contribution of each subunit to the folding cycle. Furthermore, given that some of the single point mutations in the ATP-binding pocket may also affect the hetero-oligomeric state of the whole complex, molecular dynamics (MD) simulations were employed to investigate the propensity toward the native α_8_β_8_ oligomeric state and its molecular determinants.

## MATERIALS AND METHODS

### Site-directed mutagenesis and preparation of mutants

A construct for the overexpression in *E. coli* of an 8×(His_6_)-tagged α_8_/β_8_ *Ta* thermosome (aK144HTTherm) (29) was used as a template for site-directed mutagenesis. All mutants were obtained using the Quikchange Lightning Site-Directed Mutagenesis Kit (Agilent Technologies, Santa Clara, CA, USA) according to the manufacturer’s instructions, and checked by automated sequencing. The variant constructs were used to transform *E. coli* BL21(DE3) Codon Plus-RIL cells, and the mutants expressed and purified to homogeneity as described in Paul *et al.* (29) for the wild-type (WT) thermosome. The integrity of the complexes was checked by high-resolution size-exclusion chromatography on a Superose 6 Increase column (GE Healthcare, Little Chalfont, United Kingdom) and by native-PAGE (3–12% acrylamide, molecular weight marker used: Native Marl; Life Technologies, Carlsbad, CA), and the composition in subunits was determined by Tris-Acetate SDS-PAGE (7% acrylamide, molecular weight marker used: Rainbow Broad Range MWM; Thermo Fisher Scientific, Waltham, MA) of the purified proteins. A Superose 6 column was used as a further purification step in order to isolate the hexadecameric species from smaller oligomers and isolated single subunits. All the enzymes used for nucleic acid manipulation were either from New England Biolabs (Ipswich, MA, USA) or Roche (Basel, Switzerland), and the kits used for DNA prepping and extraction/purification were from Qiagen (Hilden, Germany).

### Sequence alignment

The chaperonins sequences were aligned in Muscle 3.8 (30) *via* the resources of the European Molecular Biology Laboratory and the European Bioinformatics Institute (Cambridge, United Kingdom; *http://www.ebi.ac.uk/Tools/msa*), and are presented in BoxShade 3.21 (*http://www.ch.embnet.org/software/BOX_form.html*). The following NCBI coders refer to the aligned sequences from different species: *Ta*, α: CAC12109; and β: WP_010901684; *E. coli*, GroEL: WP_004201176; *Methanococcus maripaludis*, α: WP_011171459; *Sulfolobus solfataricus*, α: WP_009992283; β: 4XCG_B and γ: AAK43104; *S. cerevisiae*, α: NP_010498; β: NP_012124; γ: NP_012520; δ: NP_010138; ε: NP_012598; ζ: NP_010474; η: NP_012424 and θ: NP_012526.

### Modeling and molecular dynamics simulations

Molecular graphics manipulations and visualizations were performed using VMD-1.9.1 (*http://www.ks.uiuc.edu/Research/vmd/*) and Chimera-1.10.2 (31). Chimera was used to overlay subunits from the 1A6D crystal structure to generate the α_16_ and β_16_ assemblies. The Gromacs-4.6.7 (*http://www.gromacs.org*) suite of software was used to set up, energy minimize and perform the MD simulations.

Scwrl4 (32) was used to pack the side chains. Pdb2gmx was used to prepare the assemblies using the v-site hydrogen option to allow a 5-fs time step. Hydrogen atoms consistent with pH 7 were added and parameterized with the AMBER-99SB-ildn force field. Each complex was surrounded by a box 2 nm larger than the polypeptide in each dimension, and filled with TIP3P water (Transferable Intermolecular Potentials for water, 3 Points). Random water molecules were replaced by sodium and chloride ions to give a neutral (uncharged overall) box and an ionic strength of 0.15 M. Each box contained ∼880,000 atoms. Each assembly was subjected to 5000 steps of energy minimization prior to position restrained and subsequent MD simulations.

MD simulations were run for each of the WT assemblies for 50 ns in order to equilibrate the systems and data were then acquired for a further 50 ns. The ATP-binding and -hydrolysis mutations were produced in Chimera from these equilibrated assemblies, energy minimized, position restrained, and simulated for 50 ns.

All simulations were performed as NPT ensembles at 298 K using periodic boundary conditions. Short range electrostatic and van der Waals’ interactions were truncated at 1.4 nm, whereas long range electrostatics were treated with the particle-mesh Ewald’s method and a long-range dispersion correction applied. Pressure was controlled by the Berendsen barostat and temperature by the V-rescale thermostat. The simulations were integrated with a leapfrog algorithm over a 5-fs time step, constraining bond vibrations with the P-LINCS (Paralellised–LInear Constraints Solver) method. Structures were saved every 0.1 ns for analysis and after each run over 50 ns. Simulation data were accumulated on Archer, the UK National Supercomputing Service (*http://www.archer.ac.uk/*).

In each case the analyzed trajectories from the last 50 ns run were written out as Protein Data Bank (PDB) snapshots. Each subunit was then extracted from the PDB (maintaining the same coordinates) and the binding energy of the interfaces calculated using Bristol University Docking Engine (BUDE) (33). Microsoft Excel (Redmond, WA, USA) was used to graphically display the results. Images were produced with Chimera and Microsoft Paint or GIMP (GNU Image Manipulation Program, *https://www.gimp.org*/).

### Steady-state ATPase measurements

ATP hydrolysis was measured using a modification of the classic malachite green reagent (34) (Biomol Green; Enzo Life Sciences, Farmingdale, NY, USA) allowing for thermostable and reliable measurements of inorganic phosphate release at 55°C. The standard reaction buffer used in all experiments was 25 mM Tris-HCl (pH 7.5), 50 mM KCl, 20 mM MgCl_2_ and 50 mM NaCl. The reactions were started by adding the thermosome to a temperature-equilibrated reaction mixture containing the reaction buffer and ATP at increasing concentrations, in a final volume of 100 μl; all the data were collected, for a minimum of 7 min, also in the absence of thermosome in order to correct for ATP spontaneous hydrolysis at 55°C. Data for all the mutants were averaged over a minimum of 3 independent experiments, and were fitted to a standard one site Michaelis-Menten equation, assuming only one type of subunit is active at a time.

### Substrate refolding assays

The refolding yield of the recombinant substrates *Ta* rhamnose dehydrogenase (RhaD) and *Ta* aldohexose dehydrogenase was assessed as described elsewhere (29). The lag phase of the recovery in activity, not reported in the graphs for clarity, was typically 2–3 min long and was not significantly affected in any of the variants analyzed, either in the absence or presence of ATP.

## RESULTS

### Point mutations in the ATP-binding pocket influence oligomerization

**Figure 1.**
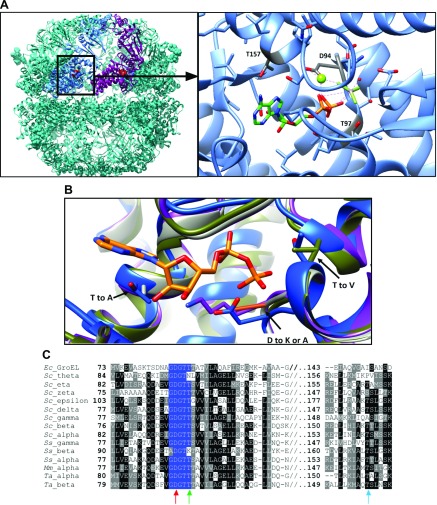
*Ta* thermosome mutants in the ATP-binding pocket of the α and β subunits. *A*) Left, side view of the ATP·AlF3–bound *Ta* thermosome (from PDB: 1A6D) with a couple of adjacent α and β subunits in light blue and magenta, respectively. The His_6_-tags protruding from each α subunit are in space-filling representation. The ATP-binding pocket of the α subunit is boxed and enlarged in the right panel, where the residues involved in ATP binding (T97 and T157) and hydrolysis (D94) are also indicated. *B*) Superposition of the WT ATP-binding pocket (blue) with that mutated at residue D93β/94α to either K or A (purple), that mutated at T96β/97α to V (green) and that mutated at T157α/158β to A (light gray). *C*) Multiple alignment of the 2 *Ta* subunits sequences with a selection of orthologous sequences; only the region of the ATP-binding pocket is shown. The mutated residues located within the region of the conserved P-loop (highlighted in blue) are indicated by the red and the green arrow (D94α/93β and T97α/96β, respectively), those outside the P-loop, T157α/158β, are indicated by the light blue arrow. *Ec*, *E. coli*; *Mm*, *M. maripaludis*; *Sc*, *S. cerevisiae* str. S288c; *Ss*, *S. solfataricus*.

**Figure 2.**
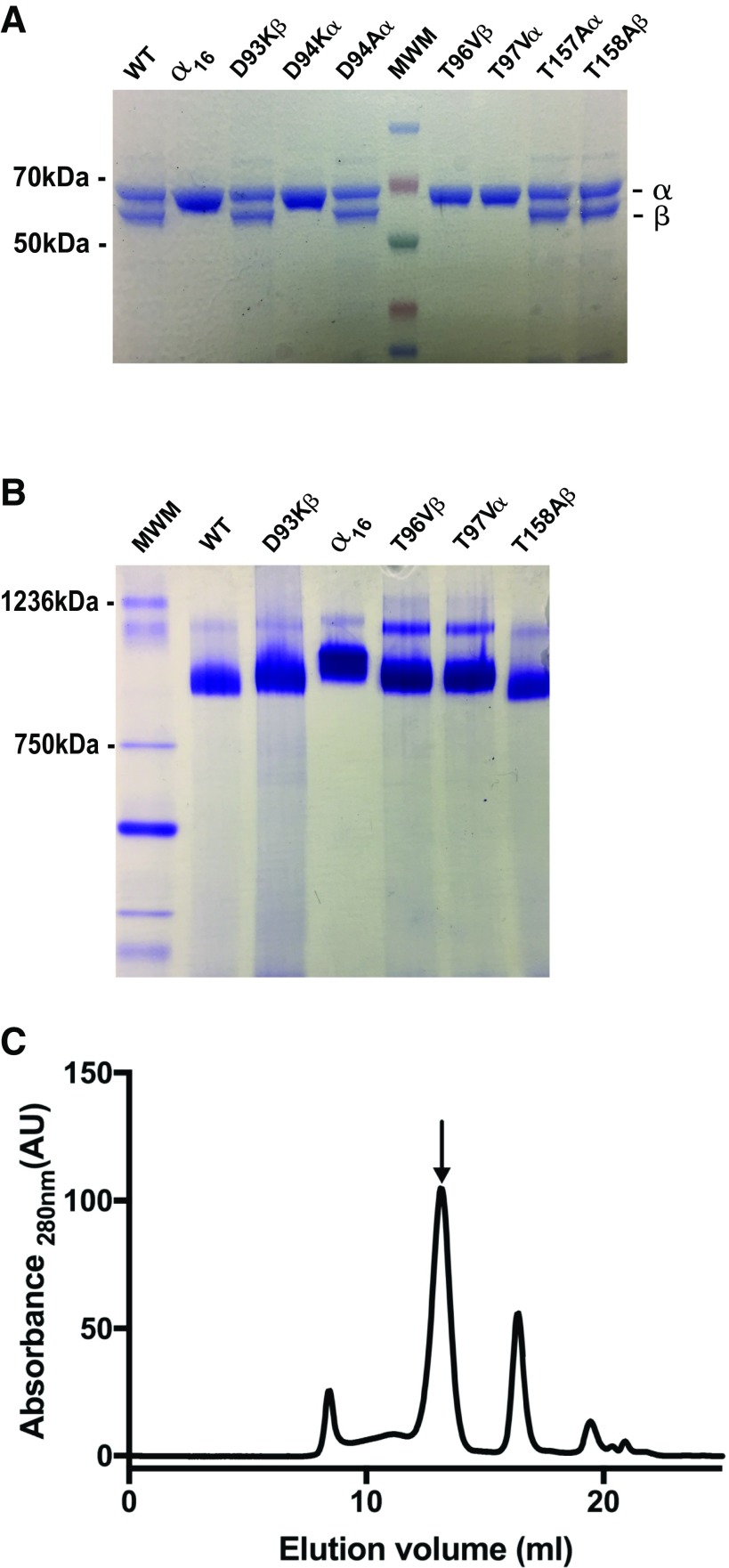
Different oligomeric states of the *Ta* thermosome mutants. *A*) SDS-PAGE of the purified mutants, identified above each lane. Masses of significant bands from the MWM (molecular weight marker) are reported on the left, the α and β bands are indicated on the right. *B*) Native-PAGE of a representative set of purified mutants, all running as ≅1 MDa complexes. MWM, molecular weight marker. *C*) Size-exclusion chromatography profile of the purified mutant D93Kβ as run on a high-resolution Superose 6 gel filtration column. The elution profiles of all the other mutants are similar, having a main peak with a retention time corresponding to a MW of ≅950 kDa (indicated by the arrow). AU, arbitrary units. The minor peaks eluting at longer retention times correspond to small α/β oligomers (mainly dimers) and single subunits; when a homogeneous population of hexadecamers was needed, it was isolated as the main peak eluting from a similar, semipreparative size-exclusion chromatography.

All the mutagenesis work was performed on a construct for the genetic manipulation and overexpression of the *Ta* thermosome previously described (29). **Figure 1*A*** shows a cartoon representation of the 8x(His_6_)-tagged hetero-oligomer thus produced. All the mutants described, either in α_8_/β_8_ or α_16_ form (**Table 1**), were purified to homogeneity following the purification protocol described for the WT protein (29) and their integrity checked by high resolution size exclusion chromatography and native PAGE (**Fig. 2*B, C***). The final yield was 5–10 mg/L bacterial culture; like the WT, all mutants, with the exception of T96Vβ and T97Vα, are remarkably stable in solution and can be stored at 4°C for days at a time.

**TABLE 1.** Steady-state ATPase and refolding activities of the *Ta* thermosome and its variants

				Refolding^*a*^ (%)	
Effect of mutation	*Ta* thermosome	Complex	ATP hydrolysis	No ATP	+ATP
	WT	α_8_β_8_	*K*_M1_ = 15 µM ± 1.65	68 ± 3.4	95 ± 2.9
			*k*_cat1_ = 2.15 ± 0.12		
			*K*_M2_ = 370 µM ± 46		
			*k*_cat2_ = 3.2 ± 0.15		
	WTα_16_	α_16_	*K*_M_= 140 µM ± 15.7	33 ± 1.7	44 ± 2
			*k*_cat_ = 0.33 ± 0.015		
ATP hydrolysis blocked	D94Kα	α_16_	None detected	21 ± 2.9	31 ± 2.6
	D94Aα	α_8_β_8_	*K*_M_ = 82 µM ± 8.7	17 ± 2.2	32 ± 2.7
			*k*_cat_ = 2.4 ± 0.09		
	D93Kβ	α_8_β_8_	*K*_M_ = 285 µM ± 35	28.5 ± 2.6	43 ± 2.8
			*k*_cat_ = 1.7 ± 0.14		
ATP binding blocked	T97Vα	α_16_	None detected	No effect	No effect
	T96Vβ	α_16_	See WTα_16_	WTα_16_	WTα_16_
	T157Aα	α_8_β_8_	None detected	24 ± 2.5	30.5 ± 2.1
	T158Aβ	α_8_β_8_	None detected	57 ± 2.2	62.6 ± 1.9

The *k*_cat_ values are expressed in moles ATP_hydr_/(mol active sites) × min. The spontaneous (*i.e.*, in the absence of thermosome) refolding yield of unfolded RhaD is 13 ± 2%. *^a^*Expressed as recovery in activity relative to the activity of the native substrate.

Based on the nucleotide-bound structure determined at high resolution by X-ray crystallography (4) or on analogies with functional mutants in GroEL (35) and yeast CCT (14), two kinds of mutations were inserted in either of the subunits in turn [*i.e.*, those abolishing ATP hydrolysis and those abolishing ATP binding altogether (Fig. 1*A, B*)]. In the former case, an aspartate residue, universally conserved in the active site of all chaperonins and shown to be involved in ATP hydrolysis both in GroEL and CCT (Fig. 1*C*), was mutated in the α and β subunits one at a time. Residues Asp93β and Asp94α (the Greek symbols identify the subunit targeted) were initially mutated to lysines, with different outcomes for the 2 subunits. The mutant D93Kβ expressed with a yield comparable to that of the WT and assembled correctly into an α_8_β_8_ hetero-oligomer. D94Kα failed to do this; the only complex that could be purified was a homo-oligomer composed of α subunits (α_16_) (Fig. 2*A, B*). The same result was obtained when mutating Asp94α to a glutamate residue (with a lower yield of intact α_16_ complex): finally, a correctly assembled α_8_β_8_ thermosome was produced with the substitution Asp94α to alanine (Fig. 2*A, B*).

Similarly, a complex and unexpected pattern emerged when mutating residues Thr96β and Thr97α, which are part of the highly conserved P loop, as first discussed by Reissmann *et al.* (15) (Fig. 1*C*). These mutations were designed to interfere with ATP binding by abolishing the stabilizing hydrogen bonds that those residues establish with the γ-phosphate of the ATP molecule in the nucleotide pocket of the β and α subunits, respectively (4) (Fig. 1). The conservative mutation of these threonines to valines in either of the subunits prevented them from assembling into α_8_β_8_ hetero-oligomers, and again only α_16_ complexes could be isolated, albeit with reduced yield and stability compared with the WT (as deduced from an increased susceptibility to proteolytic degradation during purification; data not shown). As a result, Thr157α and Thr158β were chosen as less conserved (Fig. 1*C*) and possibly less disruptive alternatives for impairing ATP binding, based on their involvement in a hydrogen bond with the nucleoside ribose in the ATP-binding pocket of the respective subunits. These mutants (T157Aα and T158Aβ) successfully formed α_8_β_8_ complexes (Fig. 2*A, B*).

### Calculation of subunit interface energies successfully predicts the *in vivo* oligomeric state

**Figure 3.**
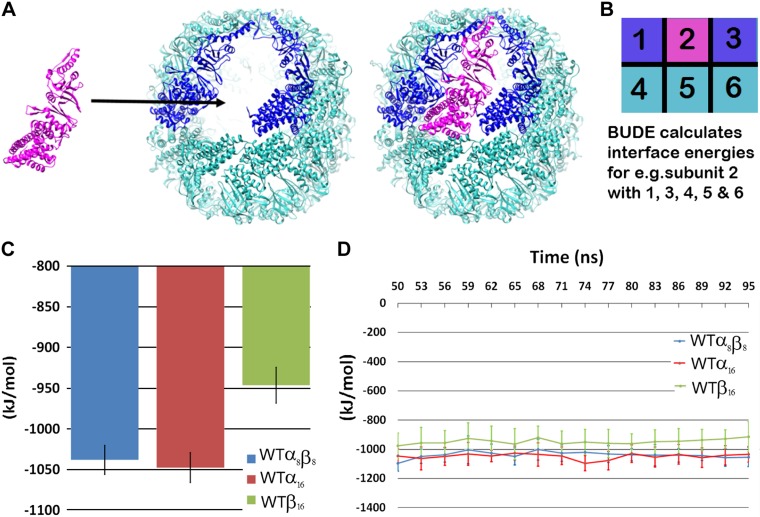
Calculation of interface energies by MD simulation and BUDE. *A*) Illustrates the calculation of the interaction energy of subunit interfaces using BUDE. *B*) Schematic example of the interfaces considered in the energy calculations for one subunit. Hence, the global energy for the subunit in position 2 was calculated using contacts made with all its neighbors 2→1, 3, 4, 5 and 6. Separate energies based on the single contributions from 1→2, 2→3 and 2→5 were also calculated and subtracted from the global energy, indicating the relative importance of the diagonal interactions 2→4 and 2→6. *C*) The calculated time point energies have been averaged for each subunit and presented graphically for the whole α_8_β_8_, α_16_ and β assemblies. *D*) BUDE-calculated global energies from structures at 3-ns intervals. The complete set of energies calculated, including those of the mutants, are reported in Table 2.

In order to investigate the determinants of the hetero-oligomeric state of the *Ta* thermosome, the first essential step was to analyze the intrinsic propensity of the WT subunits to assemble into a hetero-oligomeric complex. Experiments *in vitro* showed that when both subunits were coexpressed in *E. coli*, the α_8_β_8_ assembly formed readily. However, if the β subunits were absent or limiting, the α_16_ thermosome was formed. This is in stark contrast to the β_16_ assembly, which has not been observed experimentally either in the presence of or the absence of the α subunit. One of the possible ways to approach such complex phenomena is to use *in silico* analysis through MD simulation of the assemblies combined with assessment of the inter subunits energies using the BUDE (33) empirical free energy force field. Chaperonins are large systems, typically comprising 870,000 atoms (including explicit water), so the simulations were carried out on Archer, the UK Supercomputer, as described in Materials and Methods. The WT assemblies of the α_8_β_8_, the α_16_, and the β_16_ were simulated for 50 ns to allow the systems to equilibrate. Subsequently, each system was simulated for a further 50 ns and structures were extracted at regular intervals for interface energy analysis. From these snapshots, each subunit of each of the assemblies was analyzed for its global interface energy with respect to the rest of the thermosome (**Fig. 3*A, B***) using BUDE (36, 37). Figure 3*C* shows the global intersubunit energies, again averaged over each 50 ns trajectory, and Fig. 3*D* reports the intersubunit averaged global interface energies profile over time. The results of this analysis nicely correlate with *in vitro* experimental observations, predicting that formation of the α_8_β_8_ and α_16_ assemblies is favored over that of the β_16_.

Further calculations were performed between neighboring pairs of subunits to explore the contributions of the intra- and interring interface energies to subunit assembly as indicated in Fig. 3*A, B* and **Table 2**. These data revealed that the intraring energy contributions of the α_8_β_8_ (−450 kJ/mol) and the α_16_ (−447 kJ/mol) assemblies are comparable, whereas the β_16_ assembly’s (−401 kJ/mol) is less favorable. In contrast, the inter-ring energy of the β_16_ assembly is somewhat better than that of the α_8_β_8_ (−123 and −114 kJ/mol, respectively), but insufficient to outweigh the less favorable β_16_ intraring energy. Moreover, the diagonal interactions (*i.e.*, those of subunit 2 with subunits 4 and 6 in Fig. 3*B*; see last column in Table 2) are weak compared with those of the closer neighbors (*i.e.*, subunits 1, 3, and 5).

**TABLE 2.** BUDE-calculated interface energies for the respective assemblies

Assembly	Intraring	sd	Interring	sd	Inter-α	sd	Inter-β	sd	NRG from subs	NRG from seps	Difference
α−β											
WT^*a*^	−450	12	−114	11	−114	12	−114	9	−1038	−1013	−25
D94Aα^*a*^	−444	15	−117	9	−118	8	−115	10	−1025	−1005	−20
D93Kβ^*a*^	−443	13	−106	10	−111	8	−102	10	−1015	−992	−23
T157Aα^*a*^	−435	16	−115	11	−118	9	−111	11	−1009	−986	−23
T158Aβ^*a*^	−443	15	−111	10	−116	7	−105	10	−1022	−997	−24
T97Vα^*c*^	−438	14	−123	9	−104	10	−113	13	−1014	−999	−15
T96Vβ^*c*^	−442	12	−130	8	−106	11	−118	16	−1031	−1015	−16
D94Kα^*c*^	−440	15	−116	10	−122	7	−110	8	−1025	−997	−28
α−α											
WTα_16_^*b*^	−447	13	−137	8	−137	8	N/A	N/A	−1047	−1030	−17
D94Aα^*c*^	−453	14	−144	6	−144	6	N/A	N/A	−1048	−1050	2
D94Kα^*b*^	−445	13	−141	10	−141	10	N/A	N/A	−1065	−1031	−34
T157Aα^*c*^	−449	12	−145	8	−145	8	N/A	N/A	−1063	−1043	−20
T97Aα^*b*^	−446	14	−146	5	−146	5	N/A	N/A	−1055	−1037	−18
β−β											
WT^*c*^	−401	13	−123	5	N/A	N/A	−123	5	−946	−925	−21

All energies are expressed in kJ/mol. NRG from subs (energies from subunits) refers to the BUDE energy calculated when each subunit is docked back into the whole assembly over time points of 20-ns simulations, and then averaged over time and subunit energies. This gives an indication of strength of interaction between all the subunit interfaces, both inter- and intraring. NRG from seps refers to the BUDE energies calculated per subunit interface, separating the intraring (side-by-side) interaction energies from the interring energies designated by stacked subunits (α–α and β–β). The last column indicates the difference between the first and second method (the relative importance of the diagonal interaction in the interring interface). Footnotes denote whether the assembly is achieved *in vitro*: ^*a*^forms a–b; ^*b*^only forms a–a; ^*c*^does not form.

The ATP-binding and -hydrolysis mutants characterized in this study revealed interesting changes to the assembly propensity of thermosome molecules *in vitro* (see Table 1). The same approach was used to explore how these mutations influence subunit interface energies. Unfortunately, the signal-to-noise ratio in these experiments concealed any effect other than the consistently reduced interface energies observed for the β_16_ complexes (Supplemental Fig. S1). We suggest that the amount of sampling required by MD to attain an appropriate signal-to-noise ratio for the energy profiles is beyond current resources.

### Differential behavior of the α and β subunits toward ATP

**Figure 4.**
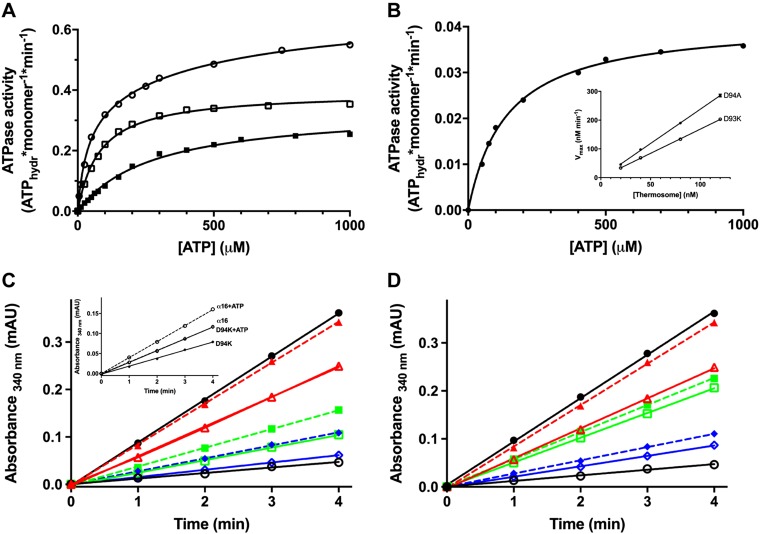
ATPase and refolding activities of the WT thermosome and its mutants. *A*) ATPase activity of the WT α_8_β_8_ thermosome (open circles) and of the hydrolysis mutants D93Kβ α_8_β_8_ (closed squares) and D94Aα α_8_β_8_ (open squares) at 55°C as a function of ATP concentration. *B*) ATPase activity of the α-only thermosome (α_16_) at 55°C as a function of ATP concentration. Inset: linear dependence of the maximum hydrolytic rate of the 2 hydrolysis mutants on thermosome concentration. *C*) Substrate refolding activity at 55°C, in the absence (empty symbols) and presence (filled symbols) of ATP, of the WT thermosome (red triangles) and of the hydrolysis mutants D93Kβ α_8_β_8_ (green squares) and D94Aα α_8_β_8_ (blue diamonds). The activity of the native substrate (T*a* RhaD) is indicated by the black closed circles, and the recovery in activity of unfolded *Ta* RhaD upon refolding in the absence of thermosome is illustrated by the black open circles. Results of such experiments in the presence of the homo-oligomeric complexes WT α_16_ and D94Kα α_16_ are reported in the inset. *D*) Substrate refolding activity at 55°C in the absence (empty symbols) and presence (filled symbols) of ATP, of the WT α_8_β_8_ thermosome (red triangles) and of the binding mutants T158Aβ α_8_β_8_ (green squares) and T157Aα α_8_β_8_ (blue diamonds). The refolding yields of the WT α_8_β_8_ thermosome and all mutants are reported in Table 1. Each data point reported is the mean of at least five independent experiments.

The variants in which ATP hydrolysis was impaired by mutation of the catalytically essential D93β or D94α residues were first analyzed by steady-state ATPase kinetics. Indeed, previous studies have established that the WT *Ta* thermosome, in both its native (27) and recombinant (22, 29) forms, shows a biphasic ATPase profile indicating the presence of 2 classes of ATP-binding sites. This reflects a negative cooperativity between rings (whereby only 1 ring is active at low ATP concentration), a differential behavior of the α and β subunits, or both. A thermally stable colorimetric essay (34) was used to record the steady-state production of inorganic phosphate upon ATP hydrolysis at 55°C in the presence of the thermosome as a function of ATP concentration; the results are plotted in **Fig. 4*A, B***. A linear dependence of the maximum hydrolytic rates on protein concentration was measured for all variants (Fig. 4*B*, inset), which allowed us to rule out the possibility of an equilibrium between heterogeneous oligomeric states with different ATPase activities. Crucially, these and all the ATPase activity experiments were performed on the hexadecameric complexes isolated by size-exclusion chromatography on a semipreparative Superose 6 column to ensure homogeneity and to exclude the presence of mixed populations of smaller oligomers or single subunits that elute at longer retention times (see Fig. 2*C*). All data were fitted to a single-site Michaelis-Menten equation, and the results are reported in Table 1. By contrast with what observed for the WT, the two single mutants analyzed here only show one phase to the saturation (Fig. 4*A*), indicative of a single class of binding sites. Furthermore, the α-only thermosome (WTα_16_) complex—obtained when the α subunit is expressed in *E. coli* in the absence of the β—shows a noncooperative single-transition ATPase profile (Fig. 4*B*), whereas the hydrolysis mutant D94Kα, which assembles only as D94Kα_16_, displays no measurable ATPase activity at all. D93Kβ (α_8_β_8_), in which ATP hydrolysis can only occur on the WTα subunits, displays a saturation midpoint comparable to that of the WT weak sites. Conversely, the apparent K_M_ for mutant D94Aα (α_8_β_8_), in which ATP hydrolysis must be assigned to the WTβ subunits, corresponds more closely (although ∼5-fold weaker) to the K_M_ calculated for the WT tight sites.

These results are mirrored by differential ATP turnover values, with the α subunits displaying a hydrolytic rate almost 2 times lower than that of the β subunits, again in line with what observed for the 2 transitions of the WT complex. Notably, when assembling into a α-only complex such as WTα_16_, the α subunits display a diminished rate of turnover despite a slightly higher affinity for ATP (Fig. 4*B*).

Somewhat surprisingly, none of the ATP-binding mutants, in either subunit, shows any detectable ATPase activity.

### Differential behavior of the α and β subunits toward nonnative substrates

We also explored the ability of the ATP-hydrolysis mutants to aid folding of a thermophilic protein substrate. Figure 4*C* reports the refolding yield of the unfolded endogenous substrate RhaD from *Ta* RhaD, shown to be an easily assayable substrate in such refolding experiments, (29) measured as recovery in enzymatic activity upon refolding in the presence of the mutant chaperonins. Specifically, the renaturation assay was initiated by extensive dilution of chemically denatured (8 M urea) *Ta* RhaD into a buffer containing an excess of either D93Kβ, D94Kα, or D94Aα, with or without ATP added. RhaD catalyzes the NADP^+^-dependent oxidation of l-rhamnose, so the recovery in activity at 55°C was monitored spectrophotometrically by the rate of increase in absorbance at 340 nm that follows the reduction of NADP^+^ to NADPH; comparison with the activity of the native enzyme provides an estimate of the refolding yield. The percentage of recovery calculated for all mutants is reported in Table 1. In the present work, substrate denaturation was induced with urea instead of the previously used GdnHCl (29). Urea was used here as a chaotropic agent in order to avoid any effects that the ionic strength of GdnHCl could exert (38, 39). The WT thermosome shows a remarkable refolding activity even in the absence of ATP, with a ∼68% yield of folded RhaD that increases to ∼100% when ATP is added to the refolding mixture. All mutants (except T97Vα) are still capable, although less than the WT, of aiding the renaturation of unfolded RhaD. The most efficient of the α_8_β_8_ mutant complexes is D93Kβ, and the data indicate that, when only the α subunit is active in hydrolyzing ATP, the thermosome ability to fold substrates is slightly less than half that of the WT; when ATP hydrolysis occurs only on the β subunit, as in D94Aα, the folding activity is further diminished. The inset in Fig. 4*C* shows the recovery in activity of unfolded RhaD in the presence of the α-only variants WTα_16_ and D94Kα α_16_. Both complexes retain a certain, albeit diminished, degree of refolding ability compared with WT α_8_β_8_, demonstrating that the β subunit is not indispensable for the *Ta* thermosome to fulfill its function.

The results of refolding assays on the ATP-binding mutants are plotted in Fig. 4*D*. Despite their similar behavior toward ATP, the 2 complexes promote renaturation to different extents, with T158Aβ displaying a refolding activity double that of T157Aα.

## DISCUSSION

We have here identified a series of residues that individually influence the oligomerization state of the *Ta* thermosome, highlighting how the composition of the ATP-binding pocket is also crucial for the correct assembly of the entire chaperonin complex. In all chaperonins, the mutations altering assembly map into one of the regions with the highest degree of sequence conservation (as reported in Fig. 1*C*)—a module within the ATP-binding pocket defined as P-loop (15)—confirming its importance both from a structural and functional point of view. Specifically, mutations in both subunits of the Asp and last Thr residue of the GDGTT motif representing the P-loop have a major effect on the formation of α_4_β_4_ rings. We used computational methods to investigate the amount of energy contributed by these residues toward the correct assembly of the oligomer. High-performance computing (Archer—UK supercomputer and Bluegem BrisSynBio) and use of v-sites in GROMACS has made exploring these large systems by all-atom MD simulations more accessible. Moreover, advances in docking software such as BUDE have allowed the contribution of interface energies for individual subunits to be calculated. Although the contributions of individual P-loop residues to the assembly propensity proved too subtle to be picked up by this technique (Supplemental Figs. S1 and S2), their application to a system as complex as the thermosome was successful in predicting the preference of its WT subunits for assembly into either an α_8_β_8_ or an α_16_ oligomer with respect to the β_16_ form (Fig. 3*A*). The latter is energetically disfavored and, to our knowledge, has never been isolated *in vivo* (22, 29). This result gives computational support to the empirical observation that, when expressed and purified separately and then mixed, the α and β subunits fail to reconstitute α_8_β_8_ complexes. The assembly of αβ is favored, as shown by the exclusive isolation of hetero-hexadecamers when the two subunits are coexpressed in equal amounts (22), and as also recently reported for TF55, the chaperonin from *Sulfolobus solfataricus* (40).

Interestingly, the interaction energies between 2 β_8_ rings were predicted to be more favorable to the hexadecamer formation than the interaction energy between 2 α_4_β_4_ rings, but this appears to be insufficient to overcome the energetic cost of assembling an all-β ring. These results reflect the necessity for each ring to incorporate α subunits in order to have folding activity. Incorporation of the β subunits, with their predicted stronger interring interactions and weaker intraring interactions, may provide an appropriate degree of flexibility essential for an efficient folding cycle. It should be noted that the strict subunit composition (α_8_β_8_) found in the thermosome from the *Ta* organism (4) is not a characteristic of all hetero-oligomeric archaeal chaperonins. In cases where 3 subunits are present, assemblies can form from a variety of subunit combinations, such that they can be individually dispensable for cell viability (40, 41). Such redundancy in subunits has been proposed as a means of adapting to different environmental conditions *via* a range of subunit arrangements.

We observed that the *Ta* WTα_16_ recombinant complex has a reduced ATPase activity not only with respect to WTα_8_β_8_ but also to the D93Kβ mutant (Fig. 4*A, B*), where only the α subunits are active. Moreover, the refolding yield of WTα_16_ is not only half of that of the WT α_8_β_8_ complex, but also comparable to that of D93Kβ both in the absence and presence of ATP (Fig. 4*C*). These results suggest that the presence of the β subunits (even when inactive) does enhance the overall hydrolysis process as well as the intrinsic folding capacity of the complex, ultimately confirming that the alternation of different subunits within a ring has a key role in the folding cycle.

**Figure 5.**
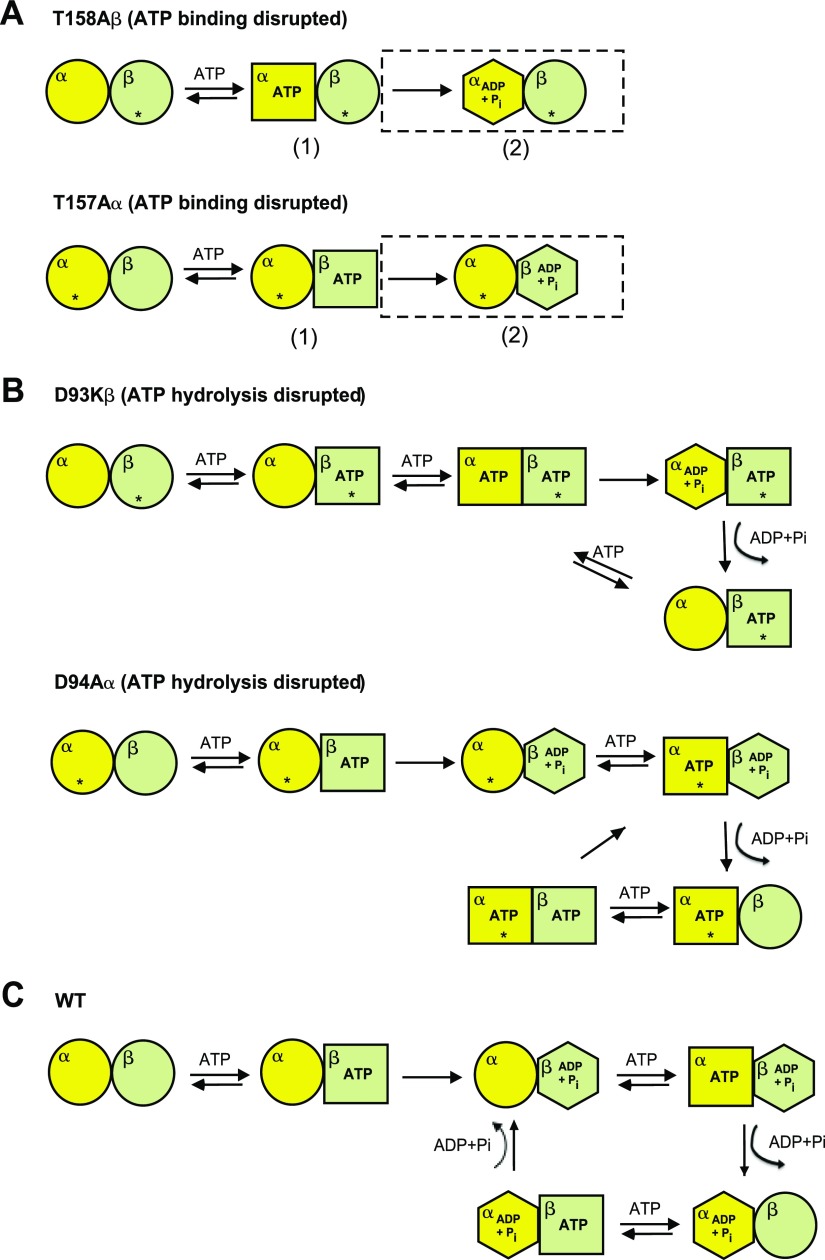
Schematics of proposed ATPase cycle of the ATP-binding (*A*) and -hydrolysis (*B*) mutants and of the WT thermosome (*C*). The circles indicate the apo state, the squares the ATP-bound state and the hexagons the posthydrolysis closed state; the asterisk marks mutated subunits. For graphical clarity, only one of the four pairs of adjacent α–β subunits per ring is displayed. *A*) Proposed rearrangements of the ATP-binding mutants. The scheme shows how the ATPase cycle in the unmodified subunit can be blocked either at the ATP-hydrolysis step (population of complex 1) or at the product release step (population of complex 2, boxed). *B*) Proposed rearrangements of the ATP-hydrolysis mutants upon ATP binding. The β (tight) subunit has a higher ATPase activity than the α (weak), but both need to bind ATP to allow cycling of the adjacent subunit. For graphical simplicity, the scheme presents only the case in which ATP binds the β subunit first, although the opposite is, in principle, possible. *C*) Proposed ATPase cycle for the WT *Ta* thermosome based on the asymmetric behavior and allostery displayed by the 2 subunits. The events are coordinated by the allosteric requirement for ATP to be bound to 1 subunit for the next one to release the hydrolysis products and be re-engaged in the cycle. Only the case in which ATP binding to 1 subunit is required for ADP and phosphate to be released from the neighboring one is reported (see Discussion for details).

The mutational analysis reported here confirms that the biphasic allosteric behavior observed in the WT *Ta* thermosome arises from the presence of two different chains that display individual characteristics with respect to ATP-binding and hydrolysis, as well as to the unfolded substrate. Although the possible contribution of the double-ring structure to asymmetry needs to be investigated, our results allow us to propose a tentative model for the interplay of events taking place in a *Ta* thermosome ring upon ATP binding and hydrolysis (**Fig. 5**). The first evidence to consider is that when ATP binding is blocked in either of the subunit types, the complex is unable to hydrolyze ATP, implying that both of the adjacent subunits need to be nucleotide-binding competent for ATP cycling to proceed at a steady state. Figure 5*A* shows the possible scenario of the cycle becoming completely blocked when the two ATP-binding mutants (T157Aα and T158Aβ) are challenged with ATP, assuming that the unmodified subunits in each case are still able to bind ATP. The scheme takes into account that the blocking of ATP binding to one subunit could impair ATP cycling in the adjacent subunit at either the stage of ATP hydrolysis (complex 1) or that of ADP+Pi release (complex 2, boxed).

Secondly, mutants with impaired ATP hydrolysis in either of the subunits (D94Aα and D93Kβ) still undergo ATP cycling in the remaining WT subunits. Interestingly, based on the complete lack of hydrolysis activity shown by the mutant α_16_ complex D94Kα, it was possible to establish that the *Ta* thermosome modification of Asp93/94 effectively abolishes ATP hydrolysis in the subunit carrying it (unlike the equivalent case for GroEL, in which hydrolysis is only partially affected) (35).

Thirdly, the kinetic constants reported in Table 1 show how the β subunit is more ATPase active (displaying a lower K_M_ and a higher *k*_cat_) than the α subunit, thus confirming that the asymmetry observed in the *Ta* thermosome cycle (22, 29) depends, at least partly, on the different contributions of ATPase activity offered by the 2 subunits. This behavior establishes a link with the eukaryotic chaperonin CCT, whose subunits have been classified as weak or strong depending on their response to ATP (with only 4 subunits at a time binding ATP in physiologic conditions in each ring) (15, 42).

It was previously reported that ATP hydrolysis in the *Ta* thermosome occurs rapidly after binding and that the rate-limiting step of the ATPase cycle is the product release of either ADP, phosphate, or both. In other words, the hydrolysis product–bound species is the most prevalent at a steady state (25). Based on all the evidence taken together, it is possible to envision a cycle in which ATP must be bound to 1 subunit for hydrolysis products to be released from the adjacent one. The scenario for the two hydrolysis mutants is shown in Fig. 5*B*, which proposes a model for a cycle carried out by the ATPase-competent subunit along with the adjacent ATPase-deficient subunit permanently loaded with ATP. For graphical clarity, the scheme only reports the cycle of events in which ATP binds to the β subunit (*i.e.*, the most ATPase active) first; although not included in the panel, a similarly asymmetric cycle comprising a complex in which the α subunit binds ATP first is also possible.

How do the observed refolding activities fit into these models? It has to be noted that the *Ta* thermosome facilitates protein folding *in vitro* to some degree, even in the absence of ATP, as do all the mutants (except the highly destabilized T97Vα). We ascribe this to its intrinsic ability to bind unfolded substrates and prevent the aggregation phenomena they would naturally undergo. It has been shown in other thermosomes that the substrate-binding sites of the apical domains, which are exposed in the apo form, are still accessible after the structural rearrangements driven by ATP binding but not after hydrolysis (43). When they are impaired in ATP binding or hydrolysis, the α or β subunits of the *Ta* thermosome are stalled in the cycle in either the apo- or ATP-bound states, respectively, maintaining substrate-binding competency.

Interestingly, ATP-binding or -hydrolysis impairment has a larger effect on folding capacity when the weak (α) subunits are targeted: although in need of further investigation, these findings could indicate an intrinsically higher affinity of the α subunits for unfolded substrates. The same phenomenon, but of opposite sign, has been observed in CCT from *S. cerevisiae*, in which ATP-hydrolysis mutants of the strong (β-equivalent) subunits were found to affect cell viability the most (14, 15). This was interpreted as a way to connect, through subunit-substrate (or substrate domain) specificity, the ATP-cycling trajectory to a specific folding pathway (17).

The asymmetry in the ATPase cycle described here points to a folding mechanism of the thermosome from *Ta* with distinct characteristics relative to that of the homo-oligomeric archaeal chaperonins, whose structural analysis by cryo-EM (6, 43, 44) and X-ray crystallography (5, 45) has been pivotal for elucidating many molecular details of the ATP-dependent behavior of class II chaperonins. Moreover, our results exclude the possibility of a concerted response to ATP, as observed in GroEL, and draw important similarities between the allosteric mechanisms of hetero-oligomeric thermosomes and eukaryotic class II chaperonins. Based on the evidence to date, a tentative model for the ATPase cycle of the WT complex is proposed in Fig. 5*C*. By maintaining next neighbor asymmetry, allosteric communication between alternating subunits with specific behaviors toward ATP allows the nonnative substrate the opportunity to remain in contact with the chaperonin, either by interacting with the apo- or ATP-bound subunits, or by having sufficient time to fold within the chamber. It may be that such asymmetric behavior could provide a way for the *Ta* thermosome to enhance and modulate its sensitivity to variations in ATP levels under different cellular conditions; this tunable behavior toward ATP might be an intrinsic property of class II chaperonins, as recently proposed by Lopez *et al.* (46). Subunit asymmetry could also reduce the need for full or synchronized lid closure, allowing the accommodation of a greater range of substrate sizes. This hypothesis is supported by the structural work of Clare *et al.* (47), who have demonstrated that the partially closed ATP-bound state, with its relatively large chamber, can accommodate substrates that would otherwise be excluded from the smaller lumen of the fully closed state.

## Supplementary Material

This article includes supplemental data. Please visit *http://www.fasebj.org* to obtain this information.

## Abbreviations

BUDE: Bristol University Docking Engine
CCT: chaperonin containing TCP-1
Cryo-EM: cryo-electron microscopy
MD: molecular dynamics
RhaD: rhamnose dehydrogenase
*Ta*: *Thermoplasma acidophilum*
WT: wild type
