# Supplementary material for: Intraring allostery controls the function and assembly of a hetero-oligomeric class II chaperonin
Source: FASEB J. 2018 Jan 5;32(4):2223–34. doi: 10.1096/fj.201701061R (PMC5983026; doi:10.1096/fj.201701061R)
Supplement: Supplementary file 1 [file fj.201701061R.sf1.docx]

**Figure S1**


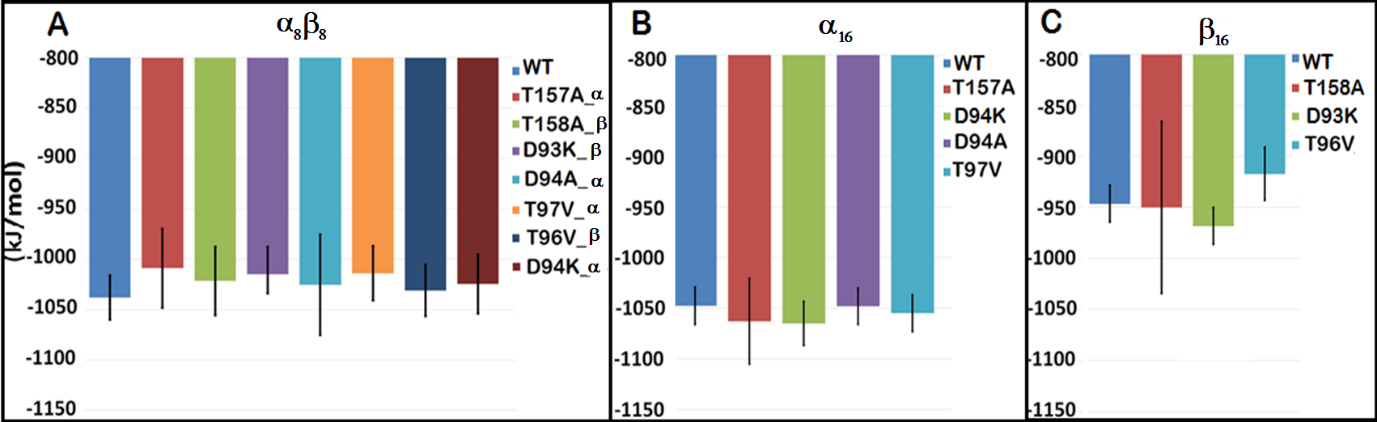


**Figure S1. Interface energies for the ATP binding and hydrolysis mutants.** Global BUDE subunit interface energies are shown averaged over all subunits and 50 ns trajectories to determine whether mutations made to disrupt ATP binding and hydrolysis affected interface energies, when compared with their wild-type equivalents. (A) BUDE-calculated global subunit interface energies for the mutations made in the α_8_β_8_ complexes. (B) BUDE-calculated global subunit interface energies for the mutations made in α_16_ assemblies. (C) BUDE-calculated global subunit interface energies for the mutations for beta_16_ assemblies.

**Figure S2**


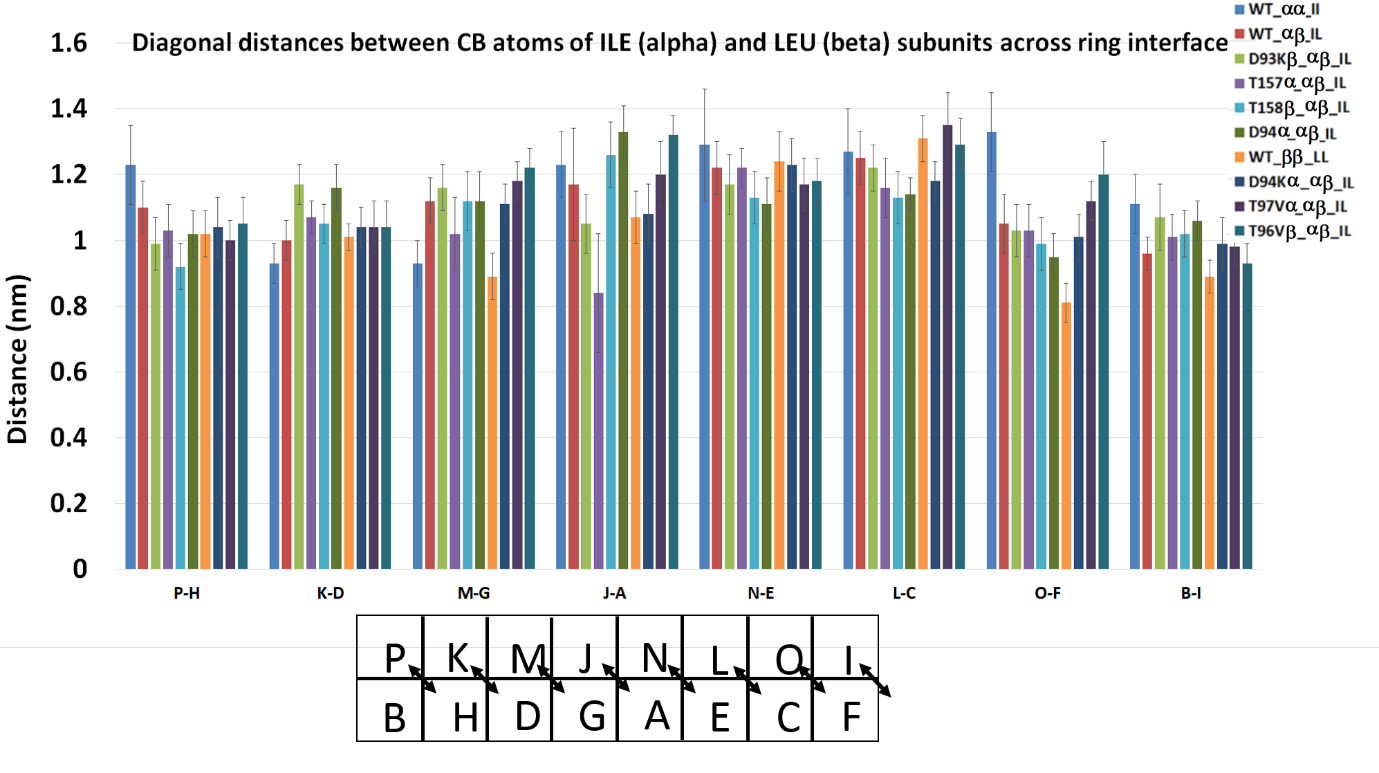


**Figure S2. Diagonal distances between subunits for the WT and mutant assemblies averaged over time.** The diagonal distances between residues that come into close contact diagonally across the ring-to-ring interface as indicated by the arrows in box, with chain labels where A,B,C,D,M,N,O&P are α subunits and E,F,G,H,I,J,K & L are β subunits in the α_8_β_8_ assemblies. IL denotes the average distances between the C-beta carbon of isoleucine sidechain in an α subunit and the C-beta of a leucine sidechain in a β subunit.
